# Psychometric properties of the Postpartum Depression Literacy Scale (PoDLiS) among Chinese perinatal women: a cross-sectional study

**DOI:** 10.1186/s12884-022-05067-2

**Published:** 2022-10-02

**Authors:** Weijian Huang, Xiaohan Li, Zijing Wu, Nan Jiang, Xu Zhang

**Affiliations:** 1 Eastern Operating Room, Yantai Yuhuangding Hospital, No.20 Yantai Yuhuangding Eastern Road, Zhifu Area, Yantai, Shandong Province P. R. China; 2 School of Nursing, China Medical University, No.77 Puhe Road, Shenyang North New Area, Shenyang, Liaoning Province P.R. China

**Keywords:** Mental health literacy, Perinatal women, Psychometric properties, Postpartum depression literacy scale

## Abstract

**Background:**

The aim of the present work was to cross-culturally adapt the Postpartum Depression Literacy Scale (PoDLiS) and use a descriptive cross-sectional design to evaluate its psychometric properties in Chinese perinatal women.

**Methods:**

Brislin’s translation theory was applied to translate the PoDLiS, with subsequent cultural adaptation. The reliability and validity of the PoDLiS were determined using a questionnaire in 635 Chinese perinatal women.

**Results:**

Good internal consistency was found (omega coefficient, 0.894) for the Chinese version of the Postpartum Depression Literacy Scale (PoDLiS-C), with omega coefficients of the six dimensions of 0.865, 0.870, 0.838, 0.777, 0.837 and 0.794, and a test–retest reliability coefficient of 0.874. The item-level content validity index (CVI) ranged from 0.8 to 1 while the scale-level CVI was 0.968. Moreover, confirmatory factor analysis (CFA) determined satisfactory construct validity of the PoDLiS-C, with the six-factor model explaining 60.76% of the total variance, demonstrating good model fit (likelihood ratio χ^2^/df, 1.003; goodness-of-fit index, 0.916; adjusted goodness-of-fit index, 0.901; comparative fit index, 0.999; incremental fit index, 0.999; root mean square error of approximation, 0.003; and standardized root mean square error of approximation, 0.0478).

**Conclusions:**

The PoDLiS-C has adequate psychometric properties. This tool could be used to assess the postpartum depression literacy of perinatal women in Chinese-speaking populations.

## Background

Postpartum depression (PPD) refers to a series of physical and psychological symptoms occurring during the perinatal period [1]. According to the DSM-V, PPD refers to a diagnosis of moderate-to-severe symptoms that occur during pregnancy or within 4 weeks of delivery. Its primary symptoms include persistent and severe depression, impaired creative thinking, lack of confidence in life, and decreased self-evaluation, as well as a series of symptoms such as anorexia, sleep disturbance, anxiety, fatigue, and even recurrent thoughts of death [2–4].

PPD has become a common perinatal complication [5], with a global prevalence of about 17% [6–8]. Furthermore, less developed countries with lower economic incomes have higher incidences of PPD [9, 10]. The incidence of PPD in Asian countries is about 21.8%, while its prevalence in China is around 27.37% and increasing annually [11–13]. The condition is now considered a public health problem that, in addition to impacting mothers and children’s health, undermines family relationships [10]. Additionally, PPD has been identified as the most disabling disease for women during the perinatal period [14].

The incidence of PPD is high, but the diagnosis rate of the disease is low, with only approximately 40% of women with PPD diagnosed [15]. PPD is very harmful, but the willingness to seek help in women with PPD is relatively low, at only 20% to 40% [16]. The majority of women do not try to get professional help to address the signs and symptoms of PPD, nor do they seek treatment for the disease. Moreover, women with PPD in less developed countries have a lower willingness to seek help [17]. Insufficient knowledge of the signs, symptoms, and treatment possibilities of PPD in perinatal women is considered to be a major obstacle in the path of women who would otherwise seek help [18, 19]. Therefore, the provision of relevant knowledge, beliefs, and attitudes is critical to help women to identify PPD and acquire efficacious treatment [18].

As defined by Jorm [20], mental health literacy (MHL) is the knowledge of and beliefs about mental illnesses that make them easy to identify, manage, or prevent. Jorm explains that MHL is not just about acquiring knowledge about mental health disorders, but is the application of that knowledge in addition to possible action to promote a person's mental health [21, 22]. MHL includes six components, namely (1) the ability to identify specific mental health conditions; (2) knowledge and beliefs of their risk factors and causes; (3) knowledge and beliefs of effective self-treatment strategies; (4) knowledge and beliefs of the available professional help and therapeutic options; (5) attitudes promoting recognition of the condition and adequate help-seeking behavior; and (6) knowledge of where to find mental health information. Most of the current assessment tools for MHL emphasize the recognition and understanding of depression [23], schizophrenia [24], and anxiety [25]. The awareness of PPD and other specific mental diseases is low.

PPD literacy assessment tools allow perinatal women and healthcare workers to understand their level of PPD literacy and to evaluate the effectiveness of various intervention strategies [26]. Using semi-structured interviews, Ransing [27] developed a 26-question, 3-point Likert scale to determine the mental health knowledge, attitudes, and awareness of a population in the perinatal period, which revealed low PPD literacy among Indian perinatal women and a misunderstanding regarding etiology and that nurse practitioner-based management may be a substantial obstacle to the delivery and utilization of PPD services. Therefore, PPD literacy must be urgently improved in perinatal women and nurse practitioners. Self-designed instruments, including the Knowledge about Postpartum Depression Questionnaire (KPPD-Q; 15 items) and the Attitudes about Postpartum Depression Questionnaire (APPD-Q; 17 items) [28] were used in the Portuguese general population. The results of the survey showed that, despite the existence of knowledge gaps and some stereotypes, the knowledge level and positive attitude of PPD were still good. A Portuguese version of the Depression Literacy Questionnaire (22 items), used to assess depression-related characteristics, identified moderate depression literacy in perinatal women [18]. Furthermore, Pamela Recto modified the Mental Health Literacy Scale (MHLS) and determined moderate PPD literacy in pregnant and postpartum Hispanic adolescents [4]. However, relatively few self-assessment tools addressing PPD in the framework of MHL have been designed. Furthermore, the inconsistency of these findings is possibly a reflection of the complexity of PPD literacy in the perinatal setting, which remains incompletely understood. Accordingly, it is meaningful to develop a PPD literacy assessment tool under the guidance of specific theory and to apply it to diverse samples.

Mirsalimi [26] developed a self-report scale to assess PPD literacy during the perinatal period (Postpartum Depression Literacy Scale [PoDLiS]), taking into account the specific aspects of MHL that have been found to become more evident in PPD while maintaining consistency with the multifaceted understanding of the theory of MHL. The PoDLiS items were built based on a review of all assessment tools for mental health literacy, qualitative research, definitions and frameworks of mental health literacy. Moreover, the PoDLiS was conceived to address critical aspects of PPD literacy. The original research, which included 692 women in the perinatal period, showed good construct and content validities and internal consistency reliability. Therefore, the scale may be used as a valuable tool for the measurement of PPD literacy levels in the perinatal period and may help us to better understand the complex mechanisms of PPD literacy.

There is no tool to measure PPD literacy in China. We thus aimed to culturally adapt and psychometrically test the PoDLiS in a Chinese sample of perinatal women.

## Materials & methods

### Setting and samples

In the current study, we used a cross-sectional design with convenience sampling. A newly developed questionnaire was implemented in two comprehensive tertiary hospitals in Yantai, China, from November 2020 to February 2021. Perinatal women were recruited from the obstetric clinic, maternity school, obstetric ward, and delivery room. According to the Kendall method for estimating sample size, we determined that the sample population would need to be from 5 to 10 times the number of items [29]. The current tool contained 31 items. Thus, considering that approximately 10% of the sample may be invalid, the required sample size was calculated to be 344 for item analysis, internal consistency and exploratory factor analysis (EFA) in the first study phase. In the second study phase, another 200 perinatal women were recruited which exceeded the minimum sample size suggested for CFA [30].

Women were considered potential participants if they a) were ≥ 20 years old; b) were currently pregnant or had given birth in the hospital participating in the study; c) had basic listening and writing skills and could read and understand the questionnaire contents on their own or with the help of others; and d) provided written informed consent. Women were excluded if they a) were undergoing in vitro fertilization; b) had severe pregnancy complications; or c) experienced stillbirths, neonatal malformations, or a baby with a serious illness. The women were explained the aims of the study and could freely refuse to participate or discontinue their participation at any time.

### Measures

#### Demographic characteristics

We collected participants’ demographic variables and those related to the pregnancy, such as age, place of residence, marital status, education, household income, perinatal period, and history of depression.

#### The Postpartum Depression Literacy Scale( PoDLiS)

The original English-language form of the PoDLiS contains 31 items and is used to measure maternal PPD literacy levels [26]. The instrument includes seven factors, namely the ability to identify PPD (six items), knowledge of risk factors and causes (five items), knowledge and beliefs of self-care activities (five items), knowledge about the availability of professional help (two items), beliefs about available professional help (two items), attitudes facilitating the recognition of PPD and appropriate help-seeking (six items), and knowledge of how to seek information related to PPD (five items). The scale uses a Likert 5-point scoring method for items 7–11 on the scale, from not likely at all (1 point) to very likely (5 points), while the other items range from strongly disagree (1 point) to strongly agree (5 points). Its reliability and validity were found to be satisfactory. For the total original PoDLiS, the Cronbach's α coefficient is 0.78 and the content validity index (CVI) is between 0.80 and 1. The construct validity was demonstrated with a χ^2^/df of 1.38, root mean square error of approximation (RMSEA) of 0.040, standardized root mean square residual (SRMR) of 0.074, comparative fit index (CFI) of 0.919, incremental fit index (IFI) of 0.921, and goodness-of-fit index (GFI) of 0.871 [26]. A higher total score on the scale represents better maternal perinatal MHL. We have permission to use this instrument from the copyright holders.

### The translation procedure

**Fig. 1:**
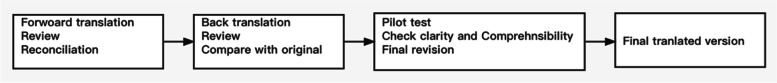
The process followed for translating and adapting the postpartum depression literacy scale for perinatal women

Authorization of the translation was obtained from the original author of the PoDLiS. We adhered to Brislin’s translation theory during the translation procedure [31] (Fig. 1). First, two bilingual postgraduate students independently translated the original scale into Chinese. Another bilingual postgraduate student separately compared and analyzed the two initial versions, consulted with the research team members to revise each item, and determined the synthesized translated version. Second, two additional translators, blinded to the original PoDLiS and with more than 1 year of overseas study experience, independently back-translated the synthesized translated version into English. Then, another bilingual English teacher, along with the research team, made a comparative analysis between the two versions and the original scale. The research team revised some wording to make the scale more culturally in line for use with a Chinese population. An incorporated back-translation version of the scale was then formed. The primary bilingual translators examined the cultural and the linguistic consistency among the back-translated version, synthesized translated version, and original English-language version, and any inconsistencies summarized in this procedure were reviewed with the author of the original version. Lastly, the synthesized version was modified to verify that the expressions maintained the original meaning.

### Expert consultations

A panel of five experts, including a psychologist, obstetrician, two head nurses of the obstetrics department, and linguist, all of whom had knowledge of PPD literacy and relevant research experience, were asked to use a 4-point Likert scale (from “not relevant” [1 point] to “highly relevant” [4 points]) to assess the degree of relevance between each item and the conceptual framework of PPD literacy and provide comments. In that process, consensus concerning the suitability and language of the synthesized version, as well as its cultural and linguistic consistency, was reached.

### Pilot testing

Thirty perinatal women were recruited to evaluate item comprehensibility. The participants required between 8 and 15 min to finish the questionnaire. The subjects thought that each item could be understood without modification. The final 31 items of our Chinese version of the PPD literacy scale (PoDLiS-C) were formed from this step and it was unanimously regarded as fluent and easy to understand.

### Data collection

Before data collection, a two-hour training session was conducted by the researcher to two research assistants who have bachelor’s degree in nursing and data collection experience. The general information about the study aims and data collection procedure was introduced. Two research assistants were trained to recruit participants and administer the instrument with supervised practices until they were competent to collect the data independently.

A printed version of the questionnaire was given to perinatal women in obstetric clinics, maternity wards, and maternity schools. Data for phase I and phase II were collected in November to December 2020 and January to February 2021, respectively. Additionally, 30 of the participants from phase I were contacted again by telephone to fill out the questionnaires to evaluate the test–retest reliability. In order to limit the women's recall of previous answers and to reduce the possibility of participants' perception change, test–retest reliability was implemented 2 weeks after their original assessment [32].

Each questionnaire was number-coded (e.g., 1, 2, 3) once all perinatal women independently completed the anonymous questionnaire. Then, the questionnaires were checked and the validity was verified. A greater than 20% missing data rate rendered a questionnaire invalid, as well as a greater than 20% missing data rate or if all options were the same.

### Psychometric testing of the scale

#### Item analysis

Item analysis was performed in accordance with the following principles: (1) extreme group comparison (meaning that items are able to discriminate between the upper 27% and lower 27% scoring groups [33]; and (2) item-total correlations (meaning that of the score of each item with the total score of the scale). We retained items that had a critical ratio greater than 3.0 or those with an item-total correlation from 0.30 to 0.80 [34].

#### Content validity

We evaluated two aspects of content validity: (1) item-level CVI (I-CVI); and (2) scale-level CVI (S-CVI). In accordance with Lynn [35], we considered an I-CVI ≥ 0.78 and an S-CVI ≥ 0.80 to be acceptable.

#### Construct validity

We used factor analysis to evaluate the construct validity of the PoDLiS-C. The normality was assessed by performing skewness and kurtosis statistics and inspecting Q-Q plot. The value of skewness less than 2 and the value of kurtosis less than 7 indicate that the data is normally distributed for the sample size larger than 300 [36]. The suitability of the factor analysis was assessed prior to the EFA using the Kaiser–Meyer–Olkin test and Bartlett’s test of sphericity. A Kaiser–Meyer–Olkin result ≥ 0.6 and a significant (*p* < 0.05) Bartlett’s test of sphericity would indicate the suitability of the scale for factor analysis [37]. Here, we divided the participants into two groups based on the data collection time for the EFA (*n* = 346) and CFA (*n* = 289).

Kaiser criterion (eigenvalue > 1.0) and parallel analysis were used to decide the number of factors to retain in EFA. Parallel analysis was more objective than scree plot and less arbitrary than Kaiser criterion (eigenvalue > 1.0) [38, 39]. Only initial eigenvalue that exceeded the eigenvalue from the parallel analysis were retained [38, 39]. Then, we performed the EFA with maximum likelihood method followed by a direct oblimin rotation to test the factor construct of all 31 items. A CFA was conducted to additionally assess the PoDLiS-C structure. The configural, measurement (in both measurement weights and measurement residuals) and structural invariance was tested based on two groups, which were participants in pregnancy period or postpartum period. The changes of CFI (△CFI) between the CFI value in unconstrained (configural) model and the CFI value for later three models less than 0.01 were acceptable [40]. The acceptable goodness-of-fit values were set at χ^2^/*df* < 3.0, RMSEA < 0.05, SRMR < 0.05, CFI > 0.9, and GFI > 0.9 [41]. Additionally, a factor loading of at least 0.3 is desirable in a CFA [30].

#### Reliability

We used McDonald’s omega coefficient to assess the internal consistency of the PoDLiS-C, which was considered satisfactory with omega coefficient exceeding 0.7 [42]. The intraclass correlation coefficient (ICC) and a two-way random model were used to assess test–retest reliability, which was considered satisfactory with an ICC exceeding 0.7.

### Data analysis

All data analyses were conducted using SPSS ver. 24.0 and AMOS ver. 24.0. Two research assistants entered data and double-checked to ensure accurate data entry. Data cleaning was performed to ensure the validity of the data analysis. The frequency counts of categorical variables were checked to detect missing data. Participants with missing responses and unanswered items were eliminated from the data analysis. Frequency and percentages were used to describe the characteristics of the demographic information of the participants, while means ± standard deviations were used to report the continuous variables. A *p* value less than 0.05 was regarded as being significant.

### Ethical considerations

Ethical approval for the study was received from the Yantai Yuhuangding Hospital Ethics Committee Ref: [2020] 283. The study was conducted according to the Declaration of Helsinki. All participants were informed of the aim of this study and had the right to refuse to participate or withdraw from the study without consequence. The written informed consent was obtained from the participants before starting the survey. All personal information and study data were kept strictly confidential. Hard copies of the data were stored in a locked cabinet, and electronic data were stored in a password-protected USB flash disk. Only the researcher could access the data.

## Results

### Characteristics of the participants

In the current study, 700 perinatal women (phase I: *n* = 400; phase II: *n* = 300) were recruited from November 2020 to February 2021, with 635 (phase I: *n* = 346; phase II: *n* = 289) completing the questionnaire according to the inclusion criteria, giving a response rate of 90.7%. The participants’ age ranged from 21 to 46 (31.79 ± 3.93) years. In general, most participants had a partner (97.5%), 69% had earned a specialty or bachelor’s degree, 78.1% were employed, 47.2% had a household monthly income > 1000 USD, 89.1% lived in urban areas, 92.3% were pregnant, and 2.7% had a history of depression. The participants’ sociodemographic characteristics are presented in Table 1.

**Table 1:** Participant characteristics (*n* = 635)

Variable	Frequency(n)/percentage(%)		
	Total (*n* = 635)	Phase I (*n* = 346)	Phase II (*n* = 289)
Age			
21–30	240 (37.8)	128 (37.0)	112 (38.8)
31–40	375 (59.1)	202 (58.4)	173 (59.9)
41–50	20 (3.1)	16 (4.6)	4 (1.4)
Marital status			
With spouse	619 (97.5)	335 (96.8)	284 (98.3)
Without spouse	16 (2.5)	11 (3.2)	5 (1.7)
Educational status			
Junior school or below	26 (4.1)	12 (3.5)	14 (4.8)
High school/specialized secondary school	107 (16.9)	57 (16.5)	50 (17.3)
Specialty/Bachelor	438 (69.0)	249 (72.0)	189 (65.4)
Postgraduate or above	64 (10.1)	28 (8.1)	36 (12.5)
Employment status			
Employed	496 (78.1)	268 (77.5)	228 (78.9)
Unemployed	139(21.9)	78 (22.5)	61 (21.1)
Household monthly income (RMB)			
< 2000	17 (2.7)	6 (1.7)	11 (3.8)
2000–4000	116 (18.3)	60 (17.3)	56 (19.4)
4001–6000	202 (31.8)	112 (32.4)	90 (31.1)
> 6000	300 (47.2)	168 (48.6)	132 (45.7)
Residence			
Urban	566 (89.1)	304 (87.9)	262 (90.7)
Rural	69 (10.9)	42 (12.1)	27 (9.3)
Current status			
Pregnancy period	576(90.7)	342 (98.8)	234 (81.0)
Postpartum period	59 (9.3)	4 (1.2)	55 (19.0)
The history of depression			
Yes	17 (2.7)	5 (1.4)	12 (4.2)
No	618 (97.3)	341 (98.6)	277 (95.8)

### Item analysis

From the extreme group comparison, the critical ratio value of the 31 items was greater than 3.0. Subsequently, Pearson’s correlation was used to determine the correlation of the items with the total score. The results showed that all item scores were positively correlated with the total score of the scale and that the item-total correlation was between 0.40 and 0.61, with a statistically significant difference (Table 2).

**Table 2:** Results of item analysis(31items)

Item	CR	Item-total correlations	Cronbach’s α if item deleted	Note
Q1	8.980*	0.552*	0.892	Retained
Q2	11.009*	0.588*	0.891	Retained
Q3	11.765*	0.576*	0.892	Retained
Q4	11.911*	0.608*	0.891	Retained
Q5	8.675*	0.609*	0.891	Retained
Q6	9.041*	0.573*	0.892	Retained
Q7	9.215*	0.469*	0.894	Retained
Q8	9.183*	0.560*	0.892	Retained
Q9	8.047*	0.556*	0.892	Retained
Q10	8.589*	0.542*	0.892	Retained
Q11	9.781*	0.572*	0.892	Retained
Q12	7.510*	0.420*	0.895	Retained
Q13	9.155*	0.566*	0.892	Retained
Q14	5.818*	0.537*	0.893	Retained
Q15	6.936*	0.490*	0.893	Retained
Q16	7.728*	0.527*	0.893	Retained
Q17	9.013*	0.532*	0.893	Retained
Q18	8.653*	0.543*	0.893	Retained
Q19	7.887*	0.447*	0.894	Retained
Q20	6.498*	0.425*	0.895	Retained
Q21	6.400*	0.430*	0.894	Retained
Q22	5.547*	0.423*	0.895	Retained
Q23	7.280*	0.435*	0.894	Retained
Q24	6.448*	0.431*	0.895	Retained
Q25	7.161*	0.446*	0.894	Retained
Q26	6.642*	0.431*	0.895	Retained
Q27	7.086*	0.410*	0.895	Retained
Q28	7.503*	0.407*	0.895	Retained
Q29	8.407*	0.434*	0.894	Retained
Q30	7.310*	0.402*	0.895	Retained
Q31	8.215*	0.408*	0.895	Retained

^*^P < 0.01

### Validity

#### Content validity

The results demonstrated that the I-CVI was between 0.8 and 1 and that the S-CVI was 0.968.

#### Construct validity

EFA was performed in the first-phase sample (*n* = 346) whose mean score of PoDLiS-C was 81.69 ± 12.80. The data satisfied the requirements of the normal distribution (skewness = 0.41 and kurtosis = 3.19). The Kaiser–Meyer–Olkin test value of 0.863 and the Bartlett record value of 5095.284 (*p* < 0.01) indicated the suitability of the PoDLiS-C for factor analysis.

By comparing with the initial eigenvalue and the eigenvalue from the parallel analysis, a six-factor structure was revealed, explaining 60.76% of the total variance. Therefore, maximum likelihood method with direct oblimin rotation was performed by extracting 6 factors (Tables 3 and 4). The extracted factors were given the following names: ability to recognize postpartum depression (Factor 1: item 1 to 6), knowledge of how to seek information related to PPD (Factor 2: item 27to 31), attitudes facilitating the identification of PPD and appropriate help-seeking (Factor 3: item 21 to 26), knowledge and beliefs concerning the available professional help for PPD (Factor 4: item 17 to 20), knowledge of PPD risk factors and causes (Factor 5: item 7 to 11), and knowledge and beliefs of PPD self-care activities (Factor 6: item 12 to 16).

**Table 3:** Pattern Matrix for the Maximum Likelihood Analysis with Direct Oblimin Rotation of the 6-Facor Solution the PoDLiS-C (*n* = 346)

Items	Factors					
	1	2	3	4	5	6
Q1 Feeling unusually sad and teary may be a symptom of postpartum depression	0.653					
Q2 Sleeping too much or too little may be a sign of postpartum depression	0.859					
Q3 Eating too much or losing appetite may be a sign of postpartum depression	0.852					
Q4 Losing interest and joy in activities may be a symptom of postpartum depression	0.660					
Q5 Postpartum depression affects person’s memory and concentration	0.391					
Q6 Symptoms and signs of postpartum depression last for at least two weeks	0.411					
Q7 How likely is postpartum depression caused by problems related to gene or heredity?					0.578	
Q8 How likely is postpartum depression caused by stressful circumstances in life (e.g. death of a family member or divorce)?					0.852	
Q9 How likely is postpartum depression caused by the lack of social support (e.g. support from intimate partner)?					0.700	
Q10 How likely is postpartum depression caused by a previous history of depression?					0.697	
Q11 How likely is postpartum depression caused by a hormonal imbalance?					0.624	
Q12 Physical activity is effective in the prevention or management of postpartum depression						0.558
Q13 Seeking help with tasks like baby care and housework from intimate partners and family members is helpful for the prevention or management of postpartum depression						0.598
Q14 Religious practices, prayer and going to church are helpful for prevention or management of postpartum depression						0.637
Q15 Balanced diet is helpful for the prevention or management of postpartum depression						0.604
Q16 Good sleep is helpful for the prevention or management of postpartum depression						0.632
Q17 Mental health professionals can treat postpartum depression effectively				0.376		
Q18 Psychotherapy (e.g. talk therapy or consultation) can effectively treat postpartum depression				0.447		
Q19 Antidepressants can be addictive				0.871		
Q20 Antidepressants can cause brain damage				0.746		
Q21 I would rather endure postpartum depression than suffer from mental treatment			0.669			
Q22 Although there are clinics for women with postpartum depression, I distrust them			0.736			
Q23 Most women with postpartum depression are violent			0.448			
Q24 It is best to avoid women with postpartum depression so that the problem will not happen to you			0.656			
Q25 If I have postpartum depression, I won’t tell anyone			0.796			
Q26 I’m worried about what my family and/or friends think about me because of my appointment in the psychology and/or psychiatric department			0.659			
Q27 I know where to find the information about postpartum depression		-0.447				
Q28 I know how to use various resources to search for information		-0.586				
Q29 I can appraise the accuracy of information about postpartum depression on the radio and television		-0.952				
Q30 I can appraise the accuracy of information about postpartum depression on the Internet		-0.940				
Q31 I can appraise the accuracy of the suggestions about postpartum depression given by friends and families		-0.782				
% of the variance	25.224	10.418	9.578	5.828	5.207	4.501
Cumulative variance	25.224	35.642	45.220	51.048	56.255	60.757

**Table 4:** Structure Matrix for the Maximum Likelihood Analysis with Direct Oblimin Rotation of the 6-Facor Solution the PoDLiS-C (*n* = 346)

Items	Factors					
	1	2	3	4	5	6
Q1 Feeling unusually sad and teary may be a symptom of postpartum depression	0.699					
Q2 Sleeping too much or too little may be a sign of postpartum depression	0.809					
Q3 Eating too much or losing appetite may be a sign of postpartum depression	0.814					
Q4 Losing interest and joy in activities may be a symptom of postpartum depression	0.734					
Q5 Postpartum depression affects person’s memory and concentration	0.603					
Q6 Symptoms and signs of postpartum depression last for at least two weeks	0.568					
Q7 How likely is postpartum depression caused by problems related to gene or heredity?					0.574	
Q8 How likely is postpartum depression caused by stressful circumstances in life (e.g. death of a family member or divorce)?					0.833	
Q9 How likely is postpartum depression caused by the lack of social support (e.g. support from intimate partner)?					0.744	
Q10 How likely is postpartum depression caused by a previous history of depression?					0.709	
Q11 How likely is postpartum depression caused by a hormonal imbalance?					0.701	
Q12 Physical activity is effective in the prevention or management of postpartum depression						0.551
Q13 Seeking help with tasks like baby care and housework from intimate partners and family members is helpful for the prevention or management of postpartum depression						0.682
Q14 Religious practices, prayer and going to church are helpful for prevention or management of postpartum depression						0.699
Q15 Balanced diet is helpful for the prevention or management of postpartum depression						0.656
Q16 Good sleep is helpful for the prevention or management of postpartum depression						0.687
Q17 Mental health professionals can treat postpartum depression effectively				0.496		
Q18 Psychotherapy (e.g. talk therapy or consultation) can effectively treat postpartum depression				0.563		
Q19 Antidepressants can be addictive				0.846		
Q20 Antidepressants can cause brain damage				0.760		
Q21 I would rather endure postpartum depression than suffer from mental treatment			0.706			
Q22 Although there are clinics for women with postpartum depression, I distrust them			0.729			
Q23 Most women with postpartum depression are violent			0.513			
Q24 It is best to avoid women with postpartum depression so that the problem will not happen to you			0.667			
Q25 If I have postpartum depression, I won’t tell anyone			0.788			
Q26 I’m worried about what my family and/or friends think about me because of my appointment in the psychology and/or psychiatric department			0.670			
Q27 I know where to find the information about postpartum depression		-0.499				
Q28 I know how to use various resources to search for information		-0.612				
Q29 I can appraise the accuracy of information about postpartum depression on the radio and television		-0.928				
Q30 I can appraise the accuracy of information about postpartum depression on the Internet		-0.917				
Q31 I can appraise the accuracy of the suggestions about postpartum depression given by friends and families		-0.768				
% of the variance	25.224	10.418	9.578	5.828	5.207	4.501
Cumulative variance	25.224	35.642	45.220	51.048	56.255	60.757

*PoDLiS-C* Chinese version of postpartum depression literacy scale

**Fig. 2:**
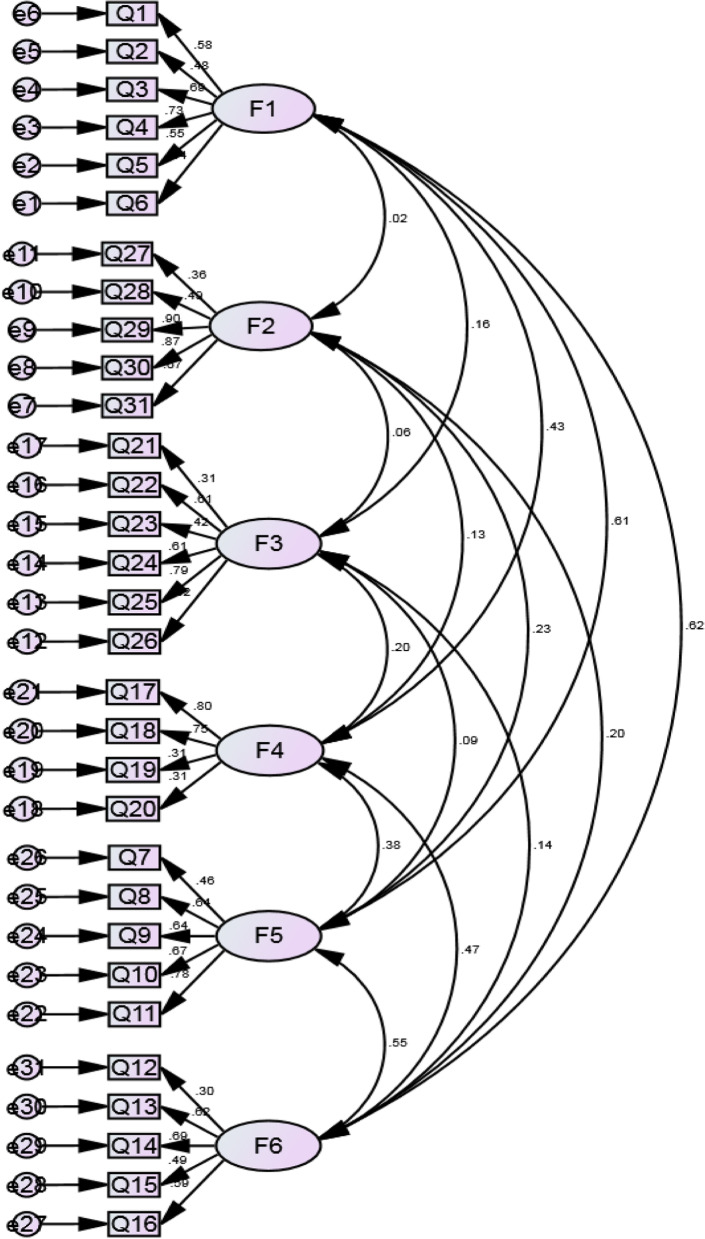
Results of confirmatory factor analysis

In accordance with the EFA results, a CFA was performed to verify the six-factor model. Compared with the one-factor model (χ^2^ = 1541.567, df = 434), the six-factor model (χ^2^ = 420.389, df = 419) appeared significant decrease of goodness-of-fit. The CFA obtained the following values: likelihood ratio (χ^2^/df), 1.003; RMSEA, 0.003; SRMR, 0.0478; GFI, 0.916; adjusted GFI, 0.901; IFI, 0.999; Tucker-Lewis index, 0.999; and CFI, 0.999 (Table 5). The CFA results suggested that the goodness-of-fit of the model of the model was acceptable (Fig. 2). As shown in Table 6, comparing with the CFI value of the unconstrained model, the changes of CFI (△CFI) in the models obtained by constraining (measurement weights, structural covariances and measurement residual) were less than 0.01. Therefore, the configural, measurement and structural invariance were ensured in this measurement model.

**Table 5:** Goodness of fit indices for the six-factor model in confirmatory factor analysis (*n* = 289)

	χ^2^	*df*	χ^2^/*df*	RMSEA	SRMR	GFI	AGFI	IFI	TLI	CFI
Criterion			< 3	< 0.05	< 0.05	> 0.90	> 0.90	> 0.90	> 0.90	> 0.90
Result	420.389	419	1.003	0.003	0.0478	0.916	0.901	0.999	0.999	0.999

*χ*^*2*^ Chi-square, *df* Degrees of freedom, *χ2/df* Normed chi-square, *RMSEA* Root mean square error of approximation, *SRMR* Standardized root mean square residual, *GFI* Goodness-of-fit index, *AGFI* Adjusted goodness-of-fit index, *IFI* Incremental fit index, *TLI* Tucker-Lewis index, *CFI* Comparative fit index

**Table 6:** Configural, measurement and structural invariance results by participants in pregnancy period or postpartum period

Model	χ^2^	*df*	χ^2^/*df*	RMSEA	SRMR	GFI	AGFI	IFI	TLI	CFI	△CFI
Unconstrained (Configural)	1053.589	838	1.257	0.030	0.0527	0.834	0.803	0.911	0.897	0.907	
Measurement weights	1078.421	863	1.250	0.029	0.0529	0.831	0.805	0.910	0.900	0.907	0.000
Structural covariances	1095.188	884	1.239	0.029	0.0540	0.831	0.810	0.911	0.904	0.909	0.002
Measurement residuals	1144.184	915	1.250	0.030	0.0545	0.819	0.804	0.902	0.900	0.901	0.006

### Reliability

The results showed the omega coefficient of the PoDLiS-C of 0.894 and obtained coefficients for the six subscales of 0.865, 0.870, 0.838, 0.777, 0.837 and 0.794, respectively (Table 7). Furthermore, the PoDLiS-C subscale internal consistency was determined to be good and not to benefit from the removal of additional items (Table 2). Therefore, all 31 items were temporarily retained. Moreover, the test–retest reliability test had a coefficient of 0.874.

**Table 7:** Results of internal consistency

Subscales	Items	Omega coefficient	Cronbach’α
1. Ability to recognize postpartum depression (item 1 to 6)	6	0.865	0.861
2. Knowledge of how to seek information related to postpartum depression (item 27 to 31)	5	0.870	0.872
3. Attitudes which facilitate recognition of postpartum depression and appropriate help-seeking (item 21 to 26)	6	0.838	0.835
4. Knowledge and beliefs about professional help available (item 17 to 20)	4	0.777	0.770
5. Knowledge of risk factors and causes (item 7 to 11)	5	0.837	0.834
6. Knowledge and beliefs of self-care activities (item 12 to 16)	5	0.794	0.791
PoDLiS-C scale	31	0.894	0.896

*PoDLis-C* The postpartum depression literacy scale-Chinese version

## Discussion

As a common psychological disorder, PPD has serious adverse effects on the mother, newborn, and family. PPD literacy is critically linked to the identification of PPD and to the help-seeking process [42]. The purpose of the current research was to translate, culturally adapt, and psychometrically evaluate the PoDLiS for China. In general, the PoDLiS-C was determined to be practicable in a domestic context and could be widely used to evaluate the PPD literacy of perinatal women.

The extreme group comparison results showed that the critical ratio values of the items ranged from 6.40 to 11.911. Thus, all were greater than 3.000 and were significant (*p* < 0.01). Moreover, the 95% confidence intervals of all items in the two groups did not include 0, indicating that the PoDLiS-C items could well identify the PPD literacy level of perinatal women. The item-total correlations were all between 0.40 and 0.61 and were significant, indicating a strong relationship to the total scale and thereby showing the relative homogeneity of these items.

In terms of content validity, the S-CVI of the PoDLiS-C was 0.968, which is higher than 0.8, and the I-CVI values were 0.8–1.0, all of which were greater than 0.78, suggesting that the content validity of the scale is satisfactory and that the item content is in good accordance with the conceptual framework. It is consistent with the content validity index of the original scale [26] and higher than the Content validity of PoDLiS in Malaysian [43] which may be related to the reason that the participates in this part are mostly pregnant women and parturients within one year after delivery, and the per capita monthly income of the family is mostly medium and high level, while the participates in Malaysia are parturients within 6 months after delivery. Household per capita monthly income is mostly associated with low and medium levels.

The seven dimensions of the original scale have not been empirically confirmed by EFA. However, the results of pattern matrix and structure matrix for the maximum likelihood analysis with direct oblimin rotation showed that the scale had six dimensions, one less than the original scale. The 7-factor model in Iran explained approximately 49% of the total variance [26], and ours comparably 60.76% of the variability. Although the factor loading value of item 5 and 17 was 0.391 and 0.376 respectively, this item was retained, given the influence of this item on the representativeness and structure of the scale.

CFA results indicated that the six-dimension structure of the PoDLiS-C yielded a largely acceptable fit for our data, with all factor loadings exceeding 0.30. Meanwhile, the dimension distribution of all items was appropriate and all items were positively related to each dimension. The six-dimension structure was more consistent with Jorm's six-factor model of MHL. This may be due to the different cultural backgrounds as well as the differences in the understanding of PPD literacy in perinatal women. Additionally, different survey samples may yield different statistical analysis results. Compared with the original research, the participants included in the current study had a higher education level and may have a stronger understanding of the value of seeking professional help. As a whole, the results of this study suggest that the PoDLiS-C model is suitable for future research work in China.

The omega coefficient was 0.894 for the overall scale and was between 0.777 and 0.870 for each subscale. These values were determined by EFA to be better than those of the original English-language scale, indicating the internal consistency reliability of the PoDLiS-C. The test–retest reliability was 0.874, showing that the stability of the PoDLiS-C was satisfactory.

The reliability and validity of the PoDLiS-C were both identified in this study. The results suggested that the scale can be used as an assessment tool for evaluating PPD literacy in Chinese perinatal women. Although the original scale has been modified in several places, all of the revisions were based on the advice of experts and were made with consideration of the Chinese cultural background. Therefore, the current instrument is more fitting for Chinese perinatal women. Community workers and healthcare providers can apply this instrument to assess the PPD literacy of perinatal women, to identify poor PPD literacy in individuals, and to design targeted interventions and public health measures in order to help to raise the public awareness of PPD and to promote the help-seeking behavior of perinatal women.

In particular, the cut-off value of the PoDLiS-C is crucial for assessing the PPD literacy of perinatal women. Future research should focus on this and develop a scientific and reasonable value to help to pinpoint women who have low PPD literacy.

### Limitations

Despite the satisfactory results, the current study has some limitations. First, the participants were recruited using convenience sampling from two hospitals of the same level in Yantai City, Shandong Province, China. Therefore, the application of these results may be limited to those who seek healthcare at hospitals of this particular level, because the PPD literacy in perinatal women may vary widely at different hospital levels. Additionally, over half of the subjects were urban dwellers and had at least a specialty or bachelor’s degree. Therefore, our results are not representative of all perinatal women in China. Second, this work was performed at a single time point. Therefore, whether the tool is able to longitudinally predict outcomes remains to be seen. Third, because dropout and responsiveness analyses were not determined, we could not summarize the characteristics of perinatal women with invalid questionnaires. Fourth, the current study evaluated the reliability and validity of the PoDLiS-C. However, further assessments of the level of PPD literacy were not conducted. In addition, there was a detailed analysis of the factors influencing the results, such as the general self-efficacy and perceived social support. Therefore, it is necessary to further verify the PoDLiS-C level in a diverse population and identify its influencing factors.

## Conclusions

To our knowledge, this is the first scale tailored to measure the PPD literacy of perinatal women in mainland China. Our findings provide evidence supporting the reliability and validity of the PoDLiS-C. Nonetheless, the PoDLiS-C and the original English scale have different factor structures. Therefore, we suggest that the sample size be increased for further reliability and validity testing. Due to the good psychometric characteristics of the scale in the current study, the PoDLiS-C has been demonstrated to be a credible and valuable instrument for enhancing the knowledge of PPD literacy as well as for boosting future investigations of PPD literacy during the perinatal period in Chinese-speaking populations.

## Data Availability

The datasets used and/or analysed during the current study available from the corresponding author on reasonable request.

## Abbreviations

PPD: Postpartum Depression
MHL: Mental Health Literacy
MHLS: Mental Health Literacy Scale
PoDLis: The Postpartum Depression Literacy Scale
PoDLiS-C: Chinese version of postpartum depression literacy scale
EFA: Exploratory factor analysis
CFA: Confirmatory factor analysis
CR: Critical ratio
I-CVI: Item-level content validity index
S-CVI: Scale-level content validity index
ICC: Intraclass correlation coefficient
RMSEA: Root mean square error of approximation
SRMR: Standardized root mean square residual
GFI: Goodness-of-fit index
AGFI: Adjusted goodness-of-fit index
IFI: Incremental fit index
TLI: Tucker-Lewis index
CFI: Comparative fit index
